# Infant inhibited temperament in primates predicts adult behavior, is heritable, and is associated with anxiety-relevant genetic variation

**DOI:** 10.1038/s41380-021-01156-4

**Published:** 2021-05-25

**Authors:** Andrew S. Fox, Ronald A. Harris, Laura Del Rosso, Muthuswamy Raveendran, Shawn Kamboj, Erin L. Kinnally, John P. Capitanio, Jeffrey Rogers

**Affiliations:** 1 Department of Psychology, University of California, Davis, CA USA; 2 California National Primate Research Center, University of California, Davis, CA USA; 3 Human Genome Sequencing Center and Department of Molecular and Human Genetics, Baylor College of Medicine, Houston, TX USA

**Keywords:** Diseases, Genetics

## Abstract

An anxious or inhibited temperament (IT) early in life is a major risk factor for the later development of stress-related psychopathology. Starting in infancy, nonhuman primates, like humans, begin to reveal their temperament when exposed to novel situations. Here, in Study 1 we demonstrate this infant IT predicts adult behavior. Specifically, in over 600 monkeys, we found that individuals scored as inhibited during infancy were more likely to refuse treats offered by potentially-threatening human experimenters as adults. In Study 2, using a sample of over 4000 monkeys from a large multi-generational family pedigree, we demonstrate that infant IT is partially heritable. The data revealed infant IT to reflect a co-inherited substrate that manifests across multiple latent variables. Finally, in Study 3 we performed whole-genome sequencing in 106 monkeys to identify IT-associated single-nucleotide variations (SNVs). Results demonstrated a genome-wide significant SNV near *CTNNA2*, suggesting a molecular target worthy of additional investigation. Moreover, we observed lower *p* values in genes implicated in human association studies of neuroticism and depression. Together, these data demonstrate the utility of our model of infant inhibited temperament in the rhesus monkey to facilitate discovery of genes that are relevant to the long-term inherited risk to develop anxiety and depressive disorders.

## Introduction

An anxious temperament (AT) or inhibited temperament (IT) early in life is a major risk factor for the later development of anxiety and depressive disorders [1–4]. Children with an extreme IT early in life are characterized by increased behavioral inhibition and anxiety in novel and potentially threatening contexts. In humans, IT reflects an inhibited nature or disposition that can define how an individual approaches novel situations throughout their life [5–7]. Although IT often emerges during the second year of life around the time that a child develops the ability to behaviorally cope with threat [8, 9] it can be preceded by increased reactivity in infancy [5]. Temperament is partially inherited, and in human samples the heritability estimates range from 20 to 50% [10–16]. Because IT is heritable, and a risk-factor for the later development of stress-related psychopathology, IT likely reflects an underlying genetic disposition and/or mediates the relationship between genetic variation and psychopathology.

To understand which molecular components contribute to anxiety and depressive disorders, researchers have begun to perform genome-wide association studies (GWAS) with dispositional anxiety and psychopathology in humans. Large-scale efforts for anxiety disorders [17, 18], depressive disorders [19, 20], and neuroticism [21] are all ongoing. Polygenic risk-scores and genetic correlation analyses, suggest each of these disorders partially overlap in their genetic substrates [17, 21]. Although some studies have identified genes with significant single-nucleotide variants (SNVs; e.g., in *CRHR1, ESR1, NTRK2, etc*.) [17, 21, 22], the specific SNVs influencing psychopathology may not be the same in individuals with different genetic backgrounds and/or who were exposed to different environmental stressors.

Rhesus macaques provide a powerful and well-validated animal model to study the genetic and molecular bases of early-life dispositional anxiety because of their similarity to humans. Next to humans, rhesus macaques are the most widely geographically distributed primates in the world, thereby exhibiting outstanding ecological flexibility and adaptability [23, 24]. As Old World monkeys, rhesus macaques are phylogenetically close to humans (with a common ancestor ~10–12 million years closer to humans than marmosets and ~50 million years closer than mice). This recent evolutionary divergence between rhesus macaques and humans is reflected in similarities in their genomes, transcript expression, brain circuits, and resulting socio-emotional behavior [25–28]. Thus, the rhesus monkey model provides a unique opportunity for translational research that supports and extends the insights gained from human genetic studies.

Capitanio et al. have developed and implemented a rhesus monkey model of infant IT, as part of their standardized BioBehavioral Assessment (BBA) that has been applied to over 4000 animals. Infant IT is characterized by below-average expression of latent variables which emerge from behavioral observations spread over 2 days, termed “Activity” and “Emotionality”, both of which are indicative of behavioral inhibition [29](see methods for details). Notably, molecular studies of dispositional anxiety in macaques are concordant with human GWAS studies, hinting at evolutionarily-conserved mechanisms (e.g., CRH [30, 31], and neurotrophic pathways [4, 32, 33]. Infant IT among macaques provides an excellent model for the study of the human risk for anxiety and depressive disorders [4, 34].

The rhesus monkey model of IT provides numerous advantages that facilitate the identification of genes and molecules that contribute to the risk for psychopathology. Rhesus populations have higher levels of genetic variation and lower linkage disequilibrium than do equivalent sample sizes from human populations, likely due to the historic population bottleneck experienced by ancient human populations that gave rise to modern humans [26, 35–38]. Importantly, this genetic variation is demonstrated in the 853 rhesus macaque whole-genome sequences that Dr. Rogers et al. have collected from US primate centers, which has identified >85 million SNVs, including 408,496 missense variants, 9921 stop codons gained and 7918 splice acceptor or donor variants [39]. Moreover, the breeding strategy at primate centers results in large multi-generational pedigrees in which each animal has many close and more distant relatives, and this will enrich the population for putatively “rare” variation, thus increasing statistical power in heritability and genetic associations analyses [40].

Here, we demonstrate that infant IT in macaques can be used as an endophenotype to complement human studies of psychopathology, and gain insight into the nature of inherited anxiety. We demonstrate that infant IT is associated with lasting behavioral changes (study 1); show that infant IT is heritable (study 2); and begin to study the genetic variation that underlie this early-life risk-factor (study 3).

## Method

### Methods overview

All studies were performed in rhesus macaques (*Macaca mulatta*) in accordance with the federal guidelines of animal use and care and with the approval of the University of California, Davis Institutional Animal Care and Use Committee. Primary analyses across all studies included animals that underwent BBA-testing during infancy (3–4 months of age). Subsets of animals were selected for Food Retrieval Task testing in Study 1 (*n* = 679; 59M/620F), heritability analyses in Study 2 (*n* = 4433; 2019M/2414F), and whole-genome sequencing in Study 3 (*n* = 106; 49M/57F). The only animals that did not undergo BBA-testing were a subset of female animals that underwent test-retest analyses in Study 1 (>2 tests: *n* = 649; >3 tests: *n* = 288; 4 tests: *n* = 88). Additional detailed methods can be found in the *Supplementary Methods* as well as previous publications [29].

### Assessment of infant IT (Studies 1–3)

The methods for scoring IT in the BBA program have been previously described [29, 34]. Animals (90–120 days old) were relocated for a 25 h testing period (see supplementary methods). IT is defined based on four factors that emerged from factor analyses in a subset of several hundred animals [29]. Animals were considered to be “inhibited” if their scores were below the mean for both “Activity” and “Emotionality” across 2 days of testing, otherwise they were classified as “not inhibited.” The “Activity” factor includes time locomoting; time NOT hanging from the top or side of the cage; rate of environmental exploration; and whether or not the animals ate food*, drank water*, or were seen crouching in the cage* (* = dichotomized due to rarity). The “Emotionality” factor includes rate of cooing; rate of barking; and whether the animals scratched*, displayed threats*, or lipsmacked* (* = dichotomized due to rarity).

### Food retrieval task

The Food Retrieval task was administered at ~6:00 am on the day after relocation, prior to morning health and husbandry, by a technician that was blind to infant IT scores. To ensure there was no familiarity with the testing context, the Food Retrieval Task was performed in a different location than BBA-testing. The technician stood in front of the cage, then approached the animal and hand presented a food treat for 5 s, taking care to avert her eyes from the monkey (a stopwatch with an audible beep was used for timing). Treat retrieval was recorded. If the animal did not accept the treat, the technician placed the treat on the forage board and stepped back from the cage, averted her eyes, waited 5 s, and again recorded whether or not the treat was retrieved. Three trials were run consecutively for each animal. Because humans in such close proximity can be perceived as threatening, the Food Retrieval task sets up a potential conflict for the animals between a fear of the human versus attraction to a favored treat.

To estimate the relationship between infant IT and refusal to take food in the food retrieval task, we used logistic regressions. To estimate test-retest stability of the dichotomous reach variable, we used chi-squre tests [41, 42]. All statistical analyses were implemented in Python (version 3.7.3), statsmodels (version 0.10.0; https://www.statsmodels.org/stable/index.html[43]; was used for regression analyses, and (Pingouin; https://pingouin-stats.org/[44]; was used to perform chi-squared tests.

### Heritability of IT

For this study, we analyzed variation among 4433 infants assessed through the BBA program between May of 2001 and January of 2017. The total sample consisted of 407 inhibited females, 403 inhibited males, 2007 non-inhibited females, and 1616 non-inhibited males. As in our previous work [45–48], all heritability and co-heritability estimates were performed using SOLAR-Eclipse (http://solar-eclipse-genetics.org/). Prior to heritability estimation phenotype variables were normalized using an inverse normal transformation. All heritability analyses controlled for sex. Because all animals were assessed between 3 and 4 months of age, we did not include Age or Age-squared as covariates in heritability analyses.

### Methods for whole-genome sequencing and mapping

Blood samples were collected from 36 inhibited animals and 70 non-inhibited animals. DNA was extracted and sequenced at the Human Genome Sequencing Center, Baylor College of Medicine using either the Illumina HiSeq 2000 or Illumina HiSeq X Ten system. WGS sequence data for the 106 animals are publicly available through the NCBI SRA (https://www.ncbi.nlm.nih.gov/biosample/?term=Bio+Behavior+Assessment). Paired end reads were aligned to the rhesus Mmul_10 reference using BWA mem with an average mapped sequence depth of 33.66X across the samples. The GATK v. 4.1.2.0 [49] pipeline was used to identify single-nucleotide variants (SNVs) and insertions/deletions (indels) smaller than 7bp. Variant Effect Predictor [50] was used to annotate variants based on merged Ensembl and RefSeq gene models.

IT-related variants were analyzed using FaST-LMM [51], which implements a linear mixed model that takes potential relatedness into account, controlling for sex. Sequence variants of interest were further examined by lifting the rhesus positions over to the orthologous human position and performing CADD analysis [52].

Permutation tests were used to compare IT-related associations to relevant gene lists extracted from published human genome-wide gene-association studies (GWGAS). First, the minimum *p* value for each gene was computed. Then the average minimum *p* value for IT-associations for each gene in the target-list was computed and stored. Finally, for each analysis we performed 10,000 permutations with a similarly sized set of randomly selected genes, and determined the average *p* value of those gene-sets. The *p* value was computed as the proportion of permutations that resulted in a lower *p* value than the target gene-set.

## Results

### Study 1: Infant IT predicts later-life behavior

**Fig. 1:**
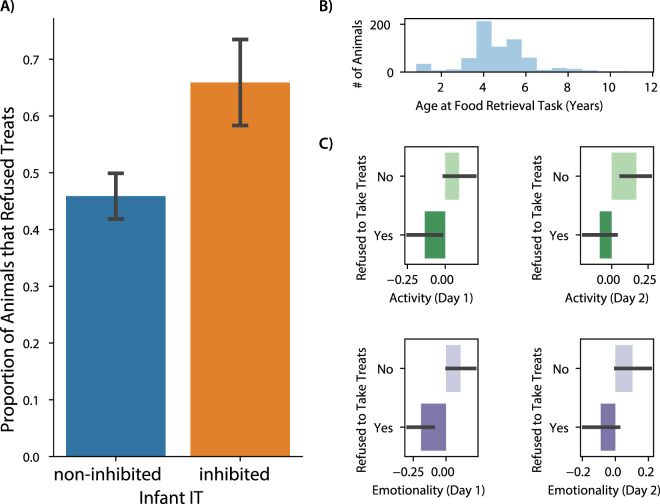
Infant Inhibited Temperament (IT) are more likely to refuse treats from a potentially-threatening human intruder. **A** Proportion of animals that refused treats in the Food Retrieval Task in relation to infant IT. **B** A histogram of ages for animals when tested in the Treat Refusal Task, on average >4 years after assessment of infant IT. **C** The factors that contribute to IT, i.e., Day 1 and 2 Activity and Emotionality, are each related to the propensity to refuse treats. Error-bars reflect bootstrapped 95% confidence interval.

To begin, we determined whether infant IT reflects an animal’s stable disposition throughout their lifespan. We assessed behavioral inhibition during a Food Retrieval Task, when adult animals (*n* = 679) were offered a treat by a human experimenter. We hypothesized that inhibited animals would forgo reward in the presence of this potentially threatening human. Logistic regressions demonstrated animals with high levels of IT at 3–4 months of age are less willing to take a treat years later (*z* = 3.248, *p* = 0.001; Fig. 1).

IT is a composite measure of two latent factors, across 2 days, i.e., Day 1 and Day 2 Activity & Emotionality. To be sure each factor of IT was contributing to the underlying temperamental variable, we assessed each variable as a predictor of treat refusal. Analyses revealed each measure, Activity and Emotionality, on each day to be predictive of treat refusal (all *p*’s < 0.014). Supplementary analyses revealed no significant effects of sex (*p* = 0.209); significant effects of IT on treat refusal in both males (*t* = 2.710, *p* = 0.007) and females (*t* = 3.193, *p* = 0.001); an effect of age on treat refusal, such that older animals were less likely to refuse a treat (*z* = −5.419, *p* < 0.001); and that IT remained significantly associated with treat refusal while controlling for age (*z* = 3.785, *p* < 0.001) (see *Supplementary Results* for additional descriptions and consideration of IT as a continuous variable). Interestingly, although animals have multiple opportunities to retrieve treats, the Food Retrieval Task typically results in an all-or-nothing result, with only ~17% (49/292) of animals who did not retrieve a treat on the first trial going on to retrieve any treat. Unsurprisingly, we obtained similar results when examining first-treat refusal, for IT (*z* = 3.248 *p* = 0.001), age (*z* = −6.064, *p* < 0.001), and IT controlling for age (2.935, *p* = 0.003). Together these data suggest that IT as assessed in this protocol is a trait-like measure, which is susceptible to change with experience, but does remain detectably consistent within an animal across contexts as they mature.

**Fig. 2:**
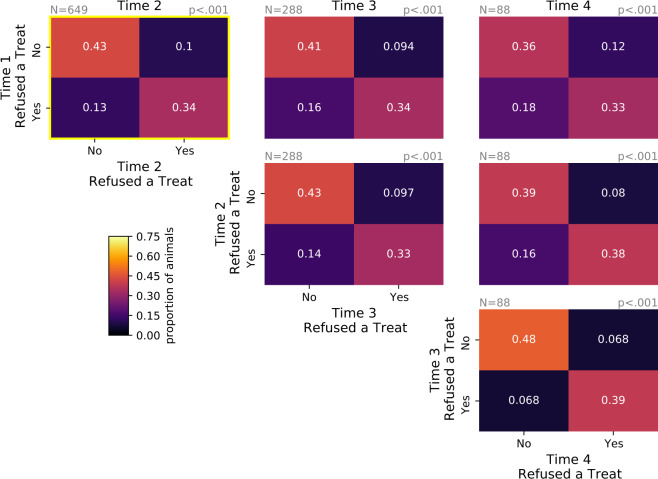
Treat refusal in the Food Retrieval Task is stable over multiple assessments. Contingency tables with the proportion of animals that refused a treat across assessments. The N and *p* value for the chi-squared test are above each contingency table in gray.

We next evaluated treat-refusal as a stable assessment. In a separate group of 649 female animals, we performed the food retrieval task multiple times, longitudinally. Animals received 2 (*N* = 649) to 4 (*N* = 88) assessments, on average 1.8 years apart. Chi-squared tests revealed treat-inhibition to be stable between each assessment (*p*’s < 0.001; Fig. 2), such that 69% of animals responded consistently during the 4th assessment on average 5.24 years later (range = 1.9–9.2 years). Again, treat refusal tended to be all-or-nothing, with 82% of animals that refused the first treat refusing all treats (chi^2^ = 430.68, *p* < 0.000001), and first-treat retrieval showing similar stability (*p*’s < 0.001).

Together, these data demonstrate infant IT reflects life-long behavioral inhibition, that reflects varied behaviors across novel contexts.

### Study 2: Infant IT is heritable

**Fig. 3:**
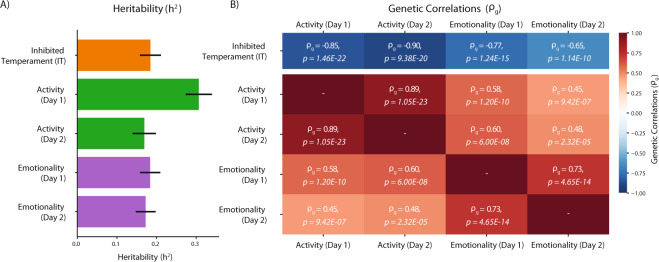
Inhibited Temperament (IT) and its latent factors are heritable, and genetically correlated. Inhibited Temperament (IT) is heritable, as are the contributing latent factors Activity and Emotionality across both days of testing (A) error-bars reflect standard deviation of the heritability estimate). In addition, IT shows a significant negative genetic correlation with each of latent factors, which in turn are positively correlated with each other (B).

We next examined the heritability of IT, and its contributing factors, across 4433 animals that were part of a large multi-generational pedigree. Results showed that IT is significantly heritable with *h*^2^ = 0.19 (std_h2_ = 0.027; *p* = 3.7e-27; Fig. 3A). Results also found IT’s latent factors, i.e., Day 1 and Day 2 Activity and Emotionality, to be significantly heritable (*p*’s < 1.0e-16) with *h*^2^ values ranging from 0.17 to 0.30. To test the extent to which the latent factors contributing to IT resulted from overlapping genetic variation, we performed genetic correlation analyses. Genetic correlation analyses test the extent to which covariation in phenotypes are associated with covariation in estimated genetic variation, as estimated by relatedness. Results demonstrated all latent factors that comprise IT were significantly genetically correlated with each other (rho-g estimates from 0.45 to 0.89; *p*’s < 0.0001) and with IT (rho-g estimates 0.65–0.90, *p*’s < 1 × 10^−9^; Fig. 3B). These data suggest a partially shared genetic substrate that contributes to these latent factors.

Together, the data from Study 2 demonstrate infant IT is significantly heritable, making it an ideal starting point to identify genetic variation. Here, we focus genetic analyses on IT which is likely to identify genetic variation that contribute to multiple latent variables, and are less likely to be specifically related to an individual feature of IT (e.g., propensity toward locomotion).

### Study 3: GWAS of IT

**Fig. 4:**
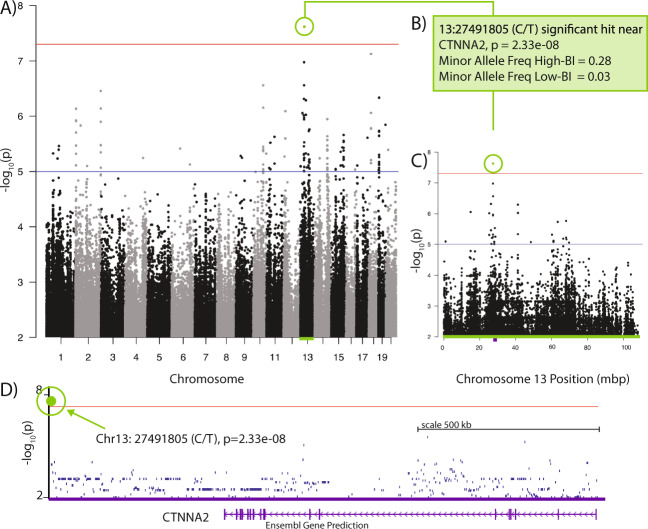
A SNV on Chromosome 13 near *CTNNA2* is associated with IT. **A** Genome-wide Manhattan plot of BioBehavioral Assessment (BBA) associations with SNVs. The *x*-axis is the chromosomes and *y*-axis is the −log_10_ of the FaST-LMM *p* value. The genome-wide significance threshold *p* < 5 × 10^−8^ is represented by the red line. The significant SNV is highlighted in green. **B** A description of the significant SNV. **C** Chromosome 13 Manhattan plot of BioBehavioral Assessment (BBA) associations with SNVs. The *x*-axis is the chromosomes and *y*-axis is the −log_10_ of the FaST-LMM *p* value. The genome-wide significance threshold *p* < 5 × 10^−8^ is represented by the red line. The significant SNV is highlighted in green. **D** UCSC Genome Browser view of SNVs with FaST-LMM −log_10_ *p* values in the vicinity of *CTNNA2*. The genome-wide significance threshold *p* < 5 × 10^−8^ is represented by the red line.

In this study, we surveyed BBA subjects for DNA sequence polymorphisms that might reasonably be hypothesized to influence IT, and determined whether the results were consistent with relevant human GWAS studies. Across the 106 individuals, we identified 53,030,128 SNVs and 6,435,882 indels using whole-genome sequencing, and used FaST-LMM to identify IT-related SNVs, taking relatedness into account. We identified a single SNV (13:27491805:C:T) that exceeded a significance threshold *p* < 5 × 10^−8^ which is a standard threshold for GWAS genome wide significance (Table S1, Fig. 4). No indels met this threshold. Two additional nearby SNVs had the third and fourth lowest *p* values in the dataset, but failed to reach the genome wide significance threshold (Table S1, Fig. 4B), including 13:27444729:G:C, 47,076bp upstream and 13:27493293:T:A, which was 1477bp downstream of the top SNV. All of these SNVs are intergenic, but 13:27491805:C:T is 473,868 bp downstream of the 3′ end of Catenin Alpha-2 (*CTNNA2*). However, although follow-up analyses revealed 4 indels and 43 SNVs in *CTNNA2*, we were unable to identify variation that was both related to IT (*p* < 0.05) and predicted to be functional (i.e., CADD > 10, see supplementary results, Tables S2–S3).

In addition to our genome-wide significant hit in *CTNNA2*, we relaxed the formal genome-wide significance threshold and explored variation in other genes that were marginally significantly (*p* < 0.01) associated with IT (Table S4), but did not reach genome-wide significance. Interestingly, our results revealed uncorrected associations with genetic variation in genes we have previously implicated in inhibition, including an 3′ UTR variant in *NTRK3* (*p* = 0.005) and an intronic variant in *PRKCD* (*p* = 0.007) [31, 33, 53]. Such associations must be considered less meaningful but can provide indications of genetic effects that deserve further study.

**Fig. 5:**
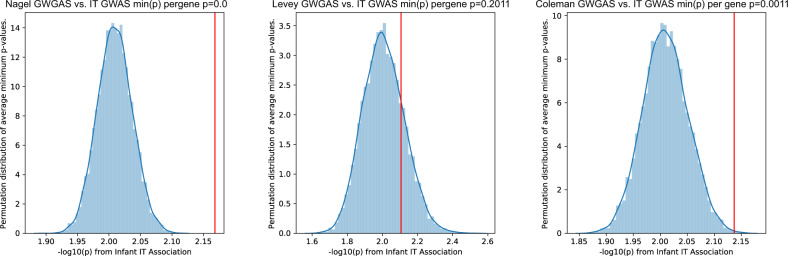
Permutation analyses of the minimum *p* value for each gene from human GWGAS studies. Permutation tests revealed the average minimum *p* value from genes found in human GWGAS studies of Neuroticism (*p* < 0.001) and Major Depressive Disorder (*p* = 0.011) was significantly lower than the average minimum *p* value in equally sized random subsets of genes. This same analysis did not reach significance for a GWGAS of Anxiety Disorders (*p* = 0.201).

Using permutation analyses, we tested for IT-related enrichment in genes relevant to stress-related psychopathology to determine if further genetic studies of monkey IT are likely to identify evolutionarily-conserved genes and pathways that are relevant to human psychopathology. Because species differences prevent base-pair by base-pair comparisons, we examined gene-level enrichment based on GWGAS from studies of human Neuroticism (547 genes; Nagel) [21], Anxiety Disorders (31 genes; Levey) [17], and Depressive Disorders (251 genes; Coleman) [22]. We examined the minimum IT-related *p* value for target genes deemed significant in published GWGAS studies. Permutation analyses found the average minimum *p* value to be significantly lower in target gene lists as compared to randomly selected genes, for neuroticism (Nagel: *p* < 0.0001) and depressive disorders (Coleman: *p* = 0.0018), but not Anxiety Disorders (Levey: *p* = 0.21; though this list only contained 31 genes) (Fig. 5). Interestingly, genes that were significant in the human GWGASs and the current analyses (*p* < 0.005, uncorrected) include *CTNNA2*, *PRKCD*, *NTRK3* and *ESR1*, highlighting the potential for identifying evolutionarily-conserved mechanisms. These data highlight the promise of our nonhuman primate approach, and suggest that additional data will identify additional IT-related genetic signals.

## Discussion

An early-life IT represents one of the strongest known risk-factors for the later development of psychopathology. Here, we demonstrate infant IT in rhesus macaques to be a heritable endophenotype that reflects stable context-independent behavior, and demonstrates promise for identifying molecules that contribute to stress-related psychopathology in humans. The data suggest infant IT is a potentially continuous measure that reflects underlying biological processes that manifest across different behaviors in different contexts (see *Supplementary Results*). These data mirror the work done in humans, in which children classified as behaviorally inhibited tend to remain inhibited as they mature, and are at a substantial risk to develop stress-related psychopathology [1–3, 54–57].

The extremely large number of phenotyped animals (*N* > 4000) that are part of a large multi-generational pedigree, provides compelling evidence for heritability, and a unique opportunity for further study of the specific SNVs that contribute to IT. Though generally consistent with our previous behavioral genetics studies of adolescent/adult rhesus macaques [30, 34, 36, 46, 58–60], previous heritability estimates tended to be slightly higher (e.g., ~25–40% heritable: [30, 45–48]. The analyses we describe here include over 4000 animals and found infant IT to be ~19% heritable (see Fig. 3). It is possible that these heritability estimates will increase as animals mature, as has been observed in twin studies of human anxiety [61, 62]. Like with human anxiety and depressive disorders, infant IT was associated with the family structure, such that inhibited animals are more likely to be related to inhibited animals.

Our preliminary genome-wide association analyses revealed a promising hit near the *CTNNA2* gene in chromosome 13, which encodes Catenin alpha-2. This is of particular interest because *CTNNA2* has been previously implicated in human association studies, including a GWGAS of anxiety disorders, and encodes a neuron-specific catenin that is important for cell-to-cell adhesion and synaptic plasticity, is expressed throughout cortical and subcortical structures, and is implicated in numerous studies of IT-related phenotypes [17, 63–70]. The association analysis presented here does not constitute evidence for a definitive association between *CTNNA2* and infant IT in the rhesus monkey. That said, together with findings in humans, these data contribute to the rationale for further study of *CTNNA2* in anxiety-related behavior in animal models, and highlight a potential molecular mechanism that may drive stable anxiety across the lifespan. More generally, our results are consistent with many molecules contributing to IT, reflecting disruption across many of the brain regions and cell-types that are thought to play a role in anxiety and threat-processing (See Supplementary discussion) [30, 32, 33, 46, 47, 53].

Moreover, these data suggest that further study of infant ITs genetics may identify molecules that are relevant to human psychopathology that can be targeted for mechanistic study. In this sample of 106 rhesus macaques, we identified IT-associations that were enriched for genes identified in GWGAS studies of ~449k subjects phenotype for neuroticism and ~624k subjects phenotyped for major depressive disorder in humans (including ~185k patients). We did not find significant enrichment for the genes implicated in a GWGAS of ~200k subjects phenotyped for anxiety disorders. This is likely because this study only identified 31 genes and will likely increase as the number of anxiety disorder genes goes up. Interestingly, although not significantly enriched for average *p* value, the *CTNNA2* gene was identified in the million veterans analysis of anxiety disorders, suggesting *CTNNA2* is a relevant gene, like CRHR1 [30], that is shared between human anxiety disorders and rhesus monkey infant-IT. Together, these data highlight the utility of using our rhesus monkey model of infant IT to identify molecules that may be relevant to human psychopathology, and suggest there is a shared molecular substrate that is conserved across primates.

## Conclusion

Elucidating the genes and genetic mechanisms that predispose individuals to stress-related psychopathology can inform our understanding of psychopathology, and guide the development of biologically-informed treatments. Understanding of disease mechanisms and developing optimal treatments requires human epidemiological insights, as well as well-validated animal models. Future studies will be needed to overcome the limitations of the current preliminary GWAS study, and increase the number of subjects to obtain additional statistical power. Importantly, with the power afforded by a pedigree-based whole-genome sequencing approach in a genetically variable species, nonhuman primate studies may be able to identify genes and specific genetic variants that would otherwise take hundreds of thousands of subjects using a traditional GWAS approach [26, 36, 38, 40]. Here, we help provide support for a translational model that can support and extend the insights gained from human genetic studies to identify the mechanisms that underlie stress-related psychopathology.

## Supplementary information

Supplemental Material

Supplemental Table 4
