# Supplementary material for: Infant inhibited temperament in primates predicts adult behavior, is heritable, and is associated with anxiety-relevant genetic variation
Source: Mol Psychiatry. 2021 May 25;26(11):6609–18. doi: 10.1038/s41380-021-01156-4 (PMC8613309; doi:10.1038/s41380-021-01156-4)
Supplement: Supplementary file 1 — Supplemental Material [file 41380_2021_1156_MOESM1_ESM.docx]

# Supplemental Materials

#### **Infant Inhibited Temperament in Primates Predicts Adult Behavior, is Heritable, and is Associated with Anxiety-Relevant Genetic Variation**

#### Authors

*Andrew S. Fox^1,2+^, *Ronald A. Harris^3^, Laura Del Rosso^2^, Muthuswamy Raveendran^3^, Shawn Kamboj S^1^, Erin L. Kinnally^1,2^, John P. Capitanio^1,2^, Jeffrey Rogers^3+^

#### Affiliations

1. Department of Psychology, University of California, Davis
2. California National Primate Research Center, University of California, Davis
3. Human Genome Sequencing Center and Department of Molecular and Human Genetics, Baylor College of Medicine, Houston, Texas

* These two authors contributed equally to this work.

+ Correspondence should be directed to Andrew Fox ([dfox@ucdavis.edu](mailto:dfox@ucdavis.edu)) and Jeffrey Rogers ([jr13@bcm.edu](mailto:jr13@bcm.edu))

####

#### Manuscript Information

**Supplemental Information:**

*Supplemental Text*: 3446

*Supplementary Tables*: 4

**Supplemental References**

**Running title:** Infant Inhibited Temperament in Primates

## Supplemental Methods

**Subjects: Overview**

All subjects were rhesus macaques (*Macaca mulatta*). Primary analyses across all studies included animals that underwent BBA testing during infancy (3-4 months of age). Subsets of animals were selected for Food Retrieval Task testing in Study 1 (n=679; 59M/620F), heritability analyses in Study 2 (n=4433; 2019M/2414F), and whole-genome sequencing in Study 3 (n=106; 49M/57F). The only animals that did not undergo BBA-testing were a subset of female animals that underwent test-retest analyses (n=649F) in Study 1. Details on each group can be found below. All studies were performed in accordance with the federal guidelines of animal use and care and with the approval of the University of California, Davis Institutional Animal Care and Use Committee.

**Assessment of Infant IT (Studies 1-3)**

Across all studies, inhibited temperament status in infant rhesus macaques was identified using the California National Primate Research Center's (CNPRC) BioBehavioral Assessment (BBA) program. This program, which has been described in detail elsewhere ([29, 34)](https://www.zotero.org/google-docs/?NBm5RJ) comprises a series of standardized behavioral and physiological assessments, conducted over a 25-hr long period when infant macaques ranged from 90-120 days of age. Assessed animals were from all four rearing environments at CNPRC: field cages, corn cribs, indoor mother reared colony, or nursery reared colony. Field cages enclosures (approximately half an acre each) house approximately 50–200 animals each. Corn cribs are roughly 400 square foot outdoor enclosures that house approximately 15–30 animals each. Field cage and corn crib reared animals were raised in their respective outdoor enclosure with their biological or foster mother. Indoor mother reared and nursery reared animals were raised indoors with no social group. Indoor mother reared animals were housed in a cage with their biological or foster mother, and at most one additional adult female and infant macaque pair. Nursery reared animals were individually housed until 3 weeks of age, at which time they were given visual access to an infant of the same age, and eventually paired with this peer at 5 weeks.

Subjects are separated from their mothers, arrive in the testing area at 0900, and are housed in standard laboratory cages (0.58 × 0.66 × 0.81 m, Lab Products, Maywood, NJ). Animals are tested in cohorts of 5-8 animals. Beginning at 0915h on Day 1, a trained observer records the behavior of each animal for five minutes using a predetermined random order and a standard ethogram for this species (see Golub et al., 2009). This assessment is repeated at 0700h the next day (Day 2). At 1000h on Day 2, infants are returned to their mothers, who had been housed in a separate area of the facility, and allowed to nurse for an hour before all animals are returned to their home cages.

As described by Golub et al. (2009) ([29)](https://www.zotero.org/google-docs/?0SR42c), behavioral data from these assessments were subjected to an exploratory factor analysis using a sample of several hundred animals. Once a factor structure was identified, a confirmatory factor analysis, involving several hundred different animals was conducted to confirm the structure. These data revealed a two factor structure that was replicable: Activity (proportion of time spent locomoting; proportion of time spent not hanging from the side of the cage, rate of environmental exploration, and dichotomous codes for whether the animal ate food, drank water, or crouched) and Emotionality (rate of cooing, rate of barking, and dichotomous codes for whether the animal scratched, displayed threats, or lipsmacked) (definitions of all behaviors are found in Golub et al. (2009) ([29)](https://www.zotero.org/google-docs/?eIk1WA). For each year, four scales were created, Activity and Emotionality for Day 1, and separately for Day 2, and values for each were z-scored with mean of 0 and standard deviation of 1. Animals were considered “inhibited” if their scores were below the mean for all four scales, and were otherwise classified as “not inhibited."

The BBA program has assessed over 5017 rhesus macaques, who are seasonal breeders, with ~78% of animals born March-May. Using the full sample, and after controlling for rearing history, birth month has a small but significant effect on the extent to which an animal is inhibited, accounting for approximately 0.3% of the variance in IT, with animals born earlier in the season slightly more likely to be BI than animals born later.

#### Study 1:

**Subjects: IT and Food Retrieval task (Study 1a)**

The subjects were 679 (n=59 male) primarily female macaques that were relocated from an outdoor enclosure (either field cages or corn cribs) for various reasons (project harvest, shipping, cage relocation etc). No animals were paired. Animals were relocated by early afternoon, and the Food Retrieval test was administered at approximately 0600h the next day, prior to morning health and husbandry. This approach minimizes any effects of separation and relocation on the animals, by leveraging separations that are a part of normal animal and veterinary care.

**Subjects: Test-Retest of Food Retrieval task (Study 1b)**

Test-retest was done on a separate group of adult female animals, many of whom did not undergo infant BBA testing. Subjects were 649 female subjects that underwent multiple food retrieval tests (at least 2 tests: n=649; at least 3 tests: n=288; 4 tests: n=88). The method for repeated assessment was identical to the initial testing.

7

**Food Retrieval Task**

The Food Retrieval task was administered at approximately 6:00 am on the day following the animal’s relocation, and prior to morning health and husbandry, by a technician that was blind to infant IT scores. In all cases, the Food Retrieval Task was performed in a different location than BBA testing, to ensure there was no familiarity with the testing context. The technician stood in front of the animal’s cage, then approached the animal and hand presented a food treat (Condition 1) for a period of five seconds, taking care to avert her eyes from the monkey (a stopwatch with an audible beep was used for timing). If the treat was taken during the five seconds, this was recorded. Any behavioral responses directed toward the technician were also recorded (data not shown). If the animal did not accept the treat, the technician took a step back, recorded the animal’s position in the cage, stepped forward, placed the treat on the forage board and stepped back from the cage (Condition 2), averted her eyes, waited five seconds, and again recorded whether or not the treat was retrieved as well as any behavioral response directed toward the technician. Three such trials were run consecutively for each animal. Because humans in such close proximity can be perceived as threatening, the Food Retrieval task sets up a potential conflict for the animals between a fear of the human versus attraction to a favored treat.

**Statistical Analyses: Food Retrieval Task**

To estimate the relationship between infant AT and refusal to take food in the food retrieval task, we used logistic regressions. To estimate test-retest stability of the dichotomous reach variable, we used chi-squre tests([41, 42)](https://www.zotero.org/google-docs/?Cn7FcH). All statistical analyses were implemented in Python (version 3.7.3), statsmodels (version 0.10.0; <https://www.statsmodels.org/stable/index.html>;([43)](https://www.zotero.org/google-docs/?KH5zvE) was used for regression analyses, and (Pingoin; <https://pingouin-stats.org/>; ([44)](https://www.zotero.org/google-docs/?IwY303) was used to perform chi-squared tests.

#### Study 2:

**Subjects: Heritability of IT**

For this study, we analyzed variation among 4433 infants (3-4 months of age) that were assessed for infant-IT as a part of the BBA program between May of 2001 and January of 2017. Parentage was assessed through breeding, blood typing, DNA. Since 1999 parentage was established with microsatellite markers([71, 72)](https://www.zotero.org/google-docs/?3NeHpq); before that, a combination of approaches were taken, including blood typing and breeding records. The total sample consisted of 407 inhibited females, 403 inhibited males, 2007 non-inhibited females, and 1616 non-inhibited males.

**Statistical Analyses: Heritability Estimation**

All heritability and bivariate heritability analyses were performed using SOLAR-Eclipse (<http://solar-eclipse-genetics.org/>). In brief, heritability is estimated using maximum likelihood, as the proportion of total of variance in an N-by-N pair-wise phenotype-covariance matrix based on the relatedness between animals ([73, 74)](https://www.zotero.org/google-docs/?vBM5f7). Prior to heritability estimation phenotype variables were normalized using an inverse normal transformation. All heritability analyses controlled for sex. Because all animals were assessed between 3-4 months of age, we did not include Age or Age-squared as covariates in heritability analyses.

Genetic correlations (𝜌_𝑔_) were estimated using bivariate heritability analyses in SOLAR-Eclipse([46, 75, 76)](https://www.zotero.org/google-docs/?XQviaZ). Bivariate heritability analyses are performed using methods similar to the heritability analyses detailed above, with a covariance matrix that represents both traits and their interaction. Significance was determined by comparing the full model to a second model in which the 𝜌_𝑔_ parameter was fixed to be 0.

#### Study 3:

**Subjects: Whole-Genome-Sequencing**

A total of 106 animals assessed for infant IT, 36 inhibited animals and 70 non-inhibited animals, were selected for whole-genome sequencing (n=49 male, n=57 female). Animal selection was based on the following criteria: field cage born and reared; reared by biological mother (ie, foster-reared animals excluded); minimally related (kinship coefficient < 0.03125); DNA available in our bank (minimal DNA for animals born in 2005-2008, so those animals were excluded).

**Methods for genome sequencing and mapping**

Rhesus macaque DNA samples (n=106) provided by Dr. John Capitanio and Dr. Erin Kinnally were sequenced at the Human Genome Sequencing Center, Baylor College of Medicine using either the Illumina HiSeq 2000 or Illumina HiSeq X Ten system. WGS sequence data for the 106 animals are publicly available through the NCBI SRA (<https://www.ncbi.nlm.nih.gov/biosample/?term=Bio+Behavior+Assessment>). The paired end reads were aligned to the rhesus Mmul_10 reference genome assembly using BWA mem with an average mapped sequence depth of 33.66X across the samples. The GATK v. 4.1.2.0 ([49)](https://www.zotero.org/google-docs/?vktGUt) pipeline was used to identify single nucleotide variants (SNVs) and insertions/deletions (indels) smaller than 7bp. Variant Effect Predictor (VEP) ([50)](https://www.zotero.org/google-docs/?hMBBtG) was used to annotate variants based on merged Ensembl and RefSeq gene models.

**Statistical Analyses: Genome-Wide Associations**

Variants were analyzed for association using FaST-LMM ([51)](https://www.zotero.org/google-docs/?d1MGGs) which implements a linear mixed model that takes into account potential relatedness among samples. FaST-LMM was run using the BioBehavioral Assessment (BBA) of inhibited (n=36) or not inhibited (n=70) as the phenotype and sex as a covariate.
 Sequence variants of interest were further examined by lifting the rhesus positions over to the orthologous human position and performing CADD ([52)](https://www.zotero.org/google-docs/?KoYEVJ) analysis which predicts the functional impact of variants. CADD integrates annotations from multiple genomic resources and calculates a score that predicts the functional impact of a variant. A CADD score of 10 indicates that the variant is predicted to be among the 10% most functional variants across the entire genome. A CADD score of 20 means that the variant falls in the top 1% most functional.

**Statistical Analyses: Permutation Analyses to Compare with Published Genome-wide gene-association studies (GWGAS)** We compared our results to 3 genome-wide gene-association studies (GWGAS) on human Neuroticism (547 genes; ([21)](https://www.zotero.org/google-docs/?Oq00lP), Anxiety Disorders (31 genes; ([17)](https://www.zotero.org/google-docs/?rggbpQ), and Depressive Disorders (251 genes; ([22)](https://www.zotero.org/google-docs/?AuXtrF). A list of relevant genes was extracted from each published GWGAS study. To perform the permutation analysis, we first computed the minimum p-value for each gene. We computed the average minimum p-value in our IT-association, in the genes reported in each list. Then, for each analysis we performed 10,000 permutations with a similarly sized set of random genes, and determined the average p-value of those gene-sets. The p-value was computed as the proportion of permutations that resulted in a lower p-value than the target gene-set.

## Supplemental Results

**Exploratory analyses for IT and behavioral inhibition in the Food Retrieval Task:**

Exploratory logistic regressions were performed to examine potentially confounding variables of sex or age during Food Retrieval Task. There were no significant effects of sex (p=.209). Additionally, IT was significantly associated with treat refusal in both males (t=2.710, p=0.007) and females (t=3.193, p=0.001), separately. There was substantial variation in the age at which animals were exposed to the Food Retrieval Test (Fig 1). Logistic regressions found that age was significantly correlated with treat refusal, such that older animals were less likely to refuse a treat (z=-5.419, p<.001). Importantly, IT remained significantly associated with treat refusal when entered simultaneously with age in a logistic regression (z=3.785, p<.001).

Interestingly, although animals have multiple opportunities to retrieve treats, the Food Retrieval Task typically results in an all-or-nothing, result, with only ~17% (49/292) of animals who did not retrieve a treat on the first trial going on to retrieve any treat. Unsurprisingly, our main results were maintained when restricting Food Retrieval Task data to refusal of the first treat, for IT (z=3.248 p=0.001), age (z=-6.064, p<.001), and IT controlling for age (2.935, p=0.003). Together these data suggest that IT as assessed in this protocol is a trait-like measure, which is susceptible to change with experience, but does remain detectably consistent within an animal across contexts as they grow up.

Variance was similar for inhibited and non-inhibited animals in the for overall treat refusal (inhibited/non-inhibited variance =.23/.25) and first treat refusal (inhibited/non-inhibited variance =.21/.25) groups.

**Exploration of IT as a continuous measure:**

The measure of infant IT that is the focus of this manuscript has previously been conceptualized as a categorical measure ([29, 77)](https://www.zotero.org/google-docs/?bIph0Z), following the J. Kagan's seminal work in humans [(78)](https://www.zotero.org/google-docs/?R8ZwR4). We sought to explore the utility of a continuous measure of IT, which would better characterize the infants in this study. To this end, we calculated a continuous measure of infant IT by taking the average of z-scored Activity and Emotionality across the two test days. Results held when using continuous infant IT as a predictor, such that it predicted treat refusal (z=4.980,p<0.001), when controlling for age (z=4.938, p<0.001), as well as in both females (z=4.436, p<0.001) and males (z=2.225, p=0.026). Importantly, when entered simultaneously into a regression with the categorical measure of IT, the continuous measure of IT remained significant (z=3.045, p=0.002). Similarly, hierarchical logistic regressions revealed that the continuous IT accounts for additional variance after accounting for the categorical measure (X^2^=9.47, p=0.002), the reverse is not true (X^2^=0.82, p=0.37). Finally, heritability analyses revealed the continuous measure of IT to have a slightly higher point-estimate of heritability than the categorical measure (22% vs. 19% heritable). Taken together, these data suggest that, if anything, the continuous measure of IT performs slightly better than the categorical measure in predicting that future studies may be better suited to use the continuous measure.

**Genome-wide significant hit and *CTNNA2:***

We searched for predicted functional variants by reciprocally lifting over each SNV to the orthologous human position and annotating each SNV with CADD information including the calculated CADD PHRED Score (Table S2). The genome-wide significant hit, 13:27491805:C:T is in an open chromatin region based on Ensembl annotations. This could have regulatory implications, but the low CADD score suggests it may not. The nearby SNV 13:27444729:G:C is annotated as intergenic, but it does have a higher CADD score (CADD=4.436). The human nucleotide orthologous to 13:27493293:T:A could not be identified with existing information and tools.

Because the variant exhibiting genome-wide significant association with IT was near *CTNNA2*, we further examined SNVs and indels in that gene without imposing a FaST-LMM p-value threshold. There were 4 indels in the UTRs of *CTNNA2*, but the most significant FaST-LMM p-value was 0.14. There were 43 SNVs in *CTNNA2* including 28 UTR variants, 14 synonymous variants and a single missense variant. The 13:28743267:C:T missense variant (Table S3) has a high CADD score of 28.5 suggesting it may have functional significance, but the p-value (p=0.48) suggests it is unlikely to have an association with IT, and the allele frequency difference between inhibited and non-inhibited animals is small. Thus, the genome-wide significant result near *CTNNA2* seems to affect IT via an unknown mechanism.

## Supplementary Discussion

Our preliminary genome-wide association analyses revealed a promising hit near the *CTNNA2* gene in chromosome 13. *CTNNA2* encodes catenin alpha-2 (also called cadherin-associated protein, alpha 2). Catenin alpha-2 is a neuron-specific catenin that is important for cell-to-cell adhesion and synaptic plasticity, and is expressed throughout cortical and subcortical structures (brain-map.org). Using a pedigree-based analysis in humans, researchers showed that biallelic loss of *CTNNA2* results in severe deficits in neuronal migration accompanied by intellectual impairment and autism-like features ([70)](https://www.zotero.org/google-docs/?GlPQaq). The only study to our knowledge to examine the mechanistic role of *CTNNA2* in fear/anxiety-related behavior found that *CTNNA2* knockout mice have a deficiency in fear-potentiated startle ([63)](https://www.zotero.org/google-docs/?QpHV89). Despite the limited understanding surrounding the function of *CTNNA2* in anxiety, numerous discovery-based analyses point to its relevance for psychopathology. Most excitingly Levey et al., (2019)([17)](https://www.zotero.org/google-docs/?cWEcY4) found SNPs in human CTNNA2 to be associated with anxiety disorders in their GWGAS (1/31 genes tested above). Consistent with a shared genetic substrate between anxiety, depression, and addiction, GWAS studies in humans have also implicated variation in the *CTNNA2* gene in impulsivity ([64)](https://www.zotero.org/google-docs/?Fn4kPH), excitement seeking ([65)](https://www.zotero.org/google-docs/?ErnicH), depression with comorbid substance abuse ([66)](https://www.zotero.org/google-docs/?NMLCQd), substance dependence ([67)](https://www.zotero.org/google-docs/?l8tpgJ), anorexia([68)](https://www.zotero.org/google-docs/?G5o4gj), and chronic pain ([69)](https://www.zotero.org/google-docs/?0YidI4). As noted in the main manuscript, the association analysis presented here does not constitute evidence for a definitive association between *CTNNA2* and infant IT in the rhesus monkey. That said, together with findings in humans, these data contribute to the rationale for further study of *CTNNA2* in anxiety-related behavior in animal models, and highlight a potential molecular mechanism that may drive stable anxiety across the lifespan.

In addition to our finding near the *CTNNA2* gene, the data point to the potential involvement of other molecular systems in infant IT. Studies in humans and nonhuman primates have identified an evolutionarily conserved distributed neural circuit that underlies anxiety and anxiety-like behavior. Importantly, this circuit includes cortical regions (e.g. orbital proviso cortex and insular regions [OPro/AI]), extended amygdala regions (e.g. central nucleus of the amygdala [Ce], and bed nucleus of the stria terminalis [BST]), as well as regions of the brainstem (e.g. periaqueductal gray [PAG]). In nonhuman primates, we have demonstrated that metabolism and connectivity across these circuits is heritable, providing a neural substrate that can mediate the effects of genes on anxiety([46, 47)](https://www.zotero.org/google-docs/?pNA4U3). We predict that the heritability and genetic effects are mediated by alterations within these circuits. For example, though it did not survive multiple comparison correction, we identified IT-related variation in *ESR1* and *NTRK2*, which have been implicated in human GWGAS studies, as well as *NTRK3* and *PRKCD*, which have been mechanistically implicated in anxiety-like behavior in animal models. These findings should be interpreted cautiously, as they did not reach formal levels of significance, but suggest a possible convergence with human genetics studies and rhesus RNA-sequencing studies that should be explored with additional statistical power.

Consistent with the results presented here, mechanistic studies in nonhuman primates and rodents have implicated specific molecules and cell-types within these regions that contribute to these distributed alterations in anxiety-related brain function. For example, we have demonstrated that expression of *NTRK3* is associated with anxiety-related responding ([32, 33)](https://www.zotero.org/google-docs/?xx6Qfm). Moreover, experimental manipulation of NTRK3-signalling in the dorsal amygdala region is sufficient to increase anxiety-related behavior in adolescent rhesus macaques([33)](https://www.zotero.org/google-docs/?lRIohZ). The data presented here, hint at additional support for the role of *NTRK3* in the risk to develop anxiety and depressive disorders by implicating a 3' variant as potentially contributing to infant IT. Along similar lines, rodent studies have identified cells expressing PKC∂ in the lateral Ce (CeL) that, when optogenetically stimulated, result in decreased freezing to a cue ([79)](https://www.zotero.org/google-docs/?RURSHq). Moreover, these neurons seem to be required for fear learning in the BLA([80)](https://www.zotero.org/google-docs/?sjemqt), and required for the effects of benzodiazepines([81)](https://www.zotero.org/google-docs/?jt3oey). These data are supported by RNA-sequencing studies in the nonhuman primate where Kovner and colleagues identified expression of the gene that encodes PKC∂, *PRKCD*, to be associated with behavioral inhibition in response to potential threat ([53)](https://www.zotero.org/google-docs/?kyN32Y). Interestingly, Kovner et al., demonstrated Ce PKC∂-expressing cells project to BST, suggesting a potential mechanism for the heritability of Ce-BST functional connectivity([48, 53)](https://www.zotero.org/google-docs/?bEwqQo). That said, again, these findings should be interpreted cautiously, and considered as modest evidence worthy of further investigation, rather than proof of these specific SNVs or genes as relevant to IT.

There are likely thousands of genes([82)](https://www.zotero.org/google-docs/?UvQ6zd) and near infinite SNVs that contribute to anxiety and depressive disorders. Identifying any single gene that is causally related to a phenotype implicitly implicates related molecules in the disorder. For example, when an individual non-synonymous SNV implicates a particular G-protein coupled receptor, this suggests the molecules that bind that receptor, regulate that receptor, or mediate its intracellular effects also may play a role in that disorder. For example, identifying BI^+^-related polymorphism in the intracellular loop of the *CRHR1* gene([30)](https://www.zotero.org/google-docs/?hekVaZ) implicates CRH ligand ([31)](https://www.zotero.org/google-docs/?TpMD53), and intracellular signaling kinases that interact with CRHR1 in the expression of anxiety. The cascade of implicated molecules does not stop there. Genes involved in causing CRH to be released and other receptors on CRH-expressing cells can also be considered likely to play a role in the risk to develop stress-related psychopathology. Although each of these implicitly related molecules is unlikely to be the cause of psychopathology in any given patient, that does not mean that they cannot be useful for treatment targets.

## Supplemental References

[71. Andrade MCR, Penedo MCT, Ward T, Silva VF, Bertolini LR, Roberts JA, et al. Determination of genetic status in a closed colony of rhesus monkeys (Macaca mulatta). Primates J Primatol. 2004;45:183–186.](https://www.zotero.org/google-docs/?Lntjfq)

[72. Kanthaswamy S, von Dollen A, Kurushima JD, Alminas O, Rogers J, Ferguson B, et al. Microsatellite markers for standardized genetic management of captive colonies of rhesus macaques (Macaca mulatta). Am J Primatol. 2006;68:73–95.](https://www.zotero.org/google-docs/?Lntjfq)

[73. Almasy L, Blangero J. Multipoint quantitative-trait linkage analysis in general pedigrees. Am J Hum Genet. 1998;62:1198–1211.](https://www.zotero.org/google-docs/?Lntjfq)

[74. Blangero J, Diego VP, Dyer TD, Almeida M, Peralta J, Kent JW, et al. A Kernel of Truth: Statistical Advances in Polygenic Variance Component Models for Complex Human Pedigrees. Adv Genet. 2013;81:1–31.](https://www.zotero.org/google-docs/?Lntjfq)

[75. Williams JT, Van Eerdewegh P, Almasy L, Blangero J. Joint Multipoint Linkage Analysis of Multivariate Qualitative and Quantitative Traits. I. Likelihood Formulation and Simulation Results. Am J Hum Genet. 1999;65:1134–1147.](https://www.zotero.org/google-docs/?Lntjfq)

[76. Almasy L, Dyer TD, Blangero J. Bivariate quantitative trait linkage analysis: pleiotropy versus co-incident linkages. Genet Epidemiol. 1997;14:953–958.](https://www.zotero.org/google-docs/?Lntjfq)

[77. Capitanio JP. Behavioral Inhibition in Nonhuman Primates: The Elephant in the Room. In: Pérez-Edgar K, Fox NA, editors. Behav. Inhib., Cham: Springer International Publishing; 2018. p. 17–33.](https://www.zotero.org/google-docs/?Lntjfq)

[78. Kagan J. Galen’s prophecy: temperament in human nature. New York: Westview Press; 1994.](https://www.zotero.org/google-docs/?Lntjfq)

[79. Haubensak W, Kunwar P, Cai H, Ciocchi S, Wall N, Ponnusamy R, et al. Genetic dissection of an amygdala microcircuit that gates conditioned fear. Nature. 2010;468:270–276.](https://www.zotero.org/google-docs/?Lntjfq)

[80. Yu K, Ahrens S, Zhang X, Schiff H, Ramakrishnan C, Fenno L, et al. The central amygdala controls learning in the lateral amygdala. Nat Neurosci. 2017;20:1680–1685.](https://www.zotero.org/google-docs/?Lntjfq)

[81. Griessner J, Pasieka M, Böhm V, Grössl F, Kaczanowska J, Pliota P, et al. Central amygdala circuit dynamics underlying the benzodiazepine anxiolytic effect. Mol Psychiatry. 2018:1–11.](https://www.zotero.org/google-docs/?Lntjfq)

[82. Flint J, Greenspan RJ, Kendler KS. How genes influence behavior. Second edition. Oxford ; New York: Oxford University Press; 2020.](https://www.zotero.org/google-docs/?Lntjfq)

# Supplemental Tables

| **SNV ID** | **Ref Allele** | **Alt Allele** | **FaST-LMM p-value** | **Inhibited Alt AF** | **Not inhibited Alt AF** |
| --- | --- | --- | --- | --- | --- |
| 13:27491805:C:T | C | T | 2.33e-08 | 0.2778 | 0.02899 |
| 13:27444729:G:C | G | C | 1.06e-07 | 0.4028 | 0.08696 |
| 13:27493293:T:A | T | A | 2.76e-07 | 0.2571 | 0.02899 |

**Table S1**. SNVs of interest based on FaST-LMM p-value including the allele nucleotides and alternate allele frequency by BBA status.

| **SNV ID** | **Human (hg38)** | **Ensembl Consequence** | **CADD Phred Score** |
| --- | --- | --- | --- |
| 13:27491805:C:T | 2:81106490 | Regulatory – Open Chromatin | 0.344 |
| 13:27444729:G:C | 2:81148697 | Intergenic | 4.436 |

**Table S2**. SNVs lifted over to the human genome and CADD results

| **SNV ID** | **Consequence** | **Ref Allele** | **Alt Allele** | **CADD Phred Score** | **FaST-LMM p-value** | **Inhibited Alt AF** | **Not inhibited Alt AF** |
| --- | --- | --- | --- | --- | --- | --- | --- |
| 13:28743267:C:T | missense | C | T | 28.5 | 0.48 | 0 | 0.0072 |

**Table S3.** *CTNNA2* functional SNVs
